# Reading Interest Profiles Among Preservice Chinese Language Teachers: Why They Begin to Like (or Dislike) Reading

**DOI:** 10.3390/bs15081111

**Published:** 2025-08-16

**Authors:** Xiaocheng Wang, Min Zhao

**Affiliations:** Department of Education, School of Humanities, Jiangnan University, Wuxi 214122, China

**Keywords:** reading interest profiles, preservice Chinese language teachers, aliteracy

## Abstract

This study aimed to examine reading interest profiles among preservice Chinese language teachers and related factors making them begin to like or dislike reading. In total, 321 college students majoring in Chinese language education in elementary and secondary schools participated in this study and completed a reading interest questionnaire. The questionnaire contains one close-ended question asking about their reading interest levels across seven periods (from preschool to college) and three open-ended questions asking about the reasons influencing their reading interest levels. Latent profile analysis (LPA) was used to identify reading interest profiles, and qualitative analysis was used to examine factors influencing their reading interests. The LPA results revealed three profiles, namely, *mountain* (up-down), *valley* (up-down-up), and *upslope* (up). The qualitative analysis revealed that motivators encouraging students to read included literacy sponsors, improved reading ability, reading time, extrinsic motivators, curiosity and desire for knowledge, access to reading, discovery of preferred texts, and relief from academic stress and relaxation. By contrast, barriers associated with the decline in reading interest included academic burdens and pressure, the availability of alternatives, a lack of reading ability, a loss of reading autonomy, a lack of literacy sponsors, limited access to reading, and inappropriate texts. Literacy researchers and educators should listen to students’ voices, understand their reading experiences, and consider developing appropriate intervention programs for literacy at different periods.

## 1. Introduction

Reading literacy is an essential skill for academic success and building a sustainable society. Both cognitive and affective factors are understood to play an essential role in reading development (Guthrie & Wigfield, 2000). However, previous research has focused on the cognitive factors of reading and attempted to determine how to teach students to read better (Jang et al., 2015). At the 1978 International Reading Convention in Houston, Texas, Larry Mikulecky directed his audience’s attention at what he believed was an emergent concern regarding literacy among US children. Often recognized as being the first to use the term *aliteracy*, Mikulecky (1978) defined it as a situation in which “capable readers are regularly choosing not to read” (p. 3), and he focused on the lack of reading habits in capable readers. For example, research found that many students stop reading when they graduate from school or only read when it is required (e.g., reading for assignments or jobs). A substantial proportion of students stop reading recreationally early in their development (Mol & Bus, 2011). One striking finding was that many Chinese adolescents do not spend time reading recreationally (Zhan et al., 2019).

As reading is an activity that requires not only skill but also the will to read, affective factors such as reading interest and enjoyment are important for students’ reading development. It is unsurprising that many studies have linked students’ reading interests to their reading performance because individuals tend to value and express interest in tasks at which they can succeed (Chapman & Tunmer, 1995). If children are interested in reading, they read more often and therefore have more opportunities to improve their reading ability than do children who prefer not to read (Kirby et al., 2011).

Unfortunately, considerable evidence shows that reading interest declines throughout the academic years (Clark, 2019; McKenna et al., 1995, 2012). Research further suggests that college students might be poorly motivated readers and functionally aliterate; that is, they can read but choose not to do so (Salter & Brook, 2007). Even more discouraging is the fact that among these college students is a significant percentage of students who aspire to become reading teachers, who will bear the responsibility of fostering deeply engaged readers (Applegate & Applegate, 2004; Applegate et al., 2014; Lee & Kim, 2022). Given the fact that teachers who are enthusiastic readers are more likely to promote students’ engagement in reading (Nathanson et al., 2008) and that teachers’ attitudes toward reading affect students’ reading enjoyment (Applegate & Applegate, 2004), understanding the reading interests and experiences of preservice language teachers is critical.

### 1.1. Reader Profiles

Given the scarcity of person-centered research on reading interest, looking closely at studies referring to reader profiles based on related factors such as reading amount is important because students with higher reading interests tend to read more (Wang et al., 2020). Park (2022) used latent profile analysis (LPA) to classify adolescent non-readers into three profiles based on their reading amount from preschool to high school: *sharply declined*, *slowly declined*, and *fixed non-readers*. Non-readers showing a sharp decline read a lot in their childhood (e.g., preschool) but stop reading from the early grades of elementary school. Non-readers showing a slow decline read a little at first, but their reading amount slowly increases until third or fourth grade, after which it once again declines. Both groups are considered *transformed* non-readers. As *non-transformed* non-readers, fixed non-readers consistently do not read from preschool to high school. In a related study, Kim (2020) examined the formation process of adult non-readers based on their reading amounts during different periods (from preschool to those in their 60s). The results indicated that high school and the years between ages 20 and 30 were typical periods of non-reader formation.

Based on the level of enjoyment associated with reading, Applegate and Applegate (2004) classified 195 preservice teachers enrolled in the initial courses of teacher certification programs in elementary education into two groups: *enthusiastic readers* (45.7%) and *unenthusiastic readers* (54.3%). Their study was replicated using a sample of 348 preservice teachers nine years later, and the number of enthusiastic readers had risen by only 2.6% to 51.1%, and nearly 40% of that gain may be accounted for by the inclusion of students preparing to teach in middle and secondary schools (Applegate et al., 2014). This is a worrying result, especially considering that many of the participants will become reading teachers who struggle to ignite in their students a love of reading that they have never experienced.

In the most relevant research, Lee and Kim (2022) examined preservice Korean language teachers’ reader profiles based on their recalled reading interest levels from preschool to college. The results revealed four profiles: *valley* (36.76%; reading interest constantly declined until college, after which it began to increase), *mountain* (26.47%; reading interest constantly increased until high school, after which it began to decline), *downslope* (26.47%; reading interest constantly declined from preschool to college), and *upslope* (10.29%; reading interest constantly increased from preschool to college). More than half the participants showed relatively low reading interest levels and ended up with declining trends in reading interest. Thus, the participants were at great risk of becoming non-readers after graduation, which deserves more attention considering the fact that most of them would become reading teachers.

### 1.2. Reading Interest and Affecting Factors

Numerous factors, such as engaging reading activities, supportive educators, and a culture that values reading (Gambrell et al., 2011), interact to shape reading interests within the school environment. Access to a wide variety of books to choose from, especially culturally relevant and high-interest materials (Luo et al., 2020) and materials tailored to meet students’ individual interests and hobbies, further amplifies intrinsic reading motivation (Guthrie et al., 2012). Peer interactions also play a pivotal role, as positive engagements among peers involving reading, book-sharing, and discussions about books can support the development and maintenance of positive attitudes toward reading (Merga, 2014).

Research has also pointed out abrupt shifts in students’ school reading experiences, such as diminished student choice, nonpersonal response expectations, detachment of reading instruction from the content, isolation of students from teachers, and estrangement of reading from real-world interactions, all of which might account for the decline in reading interests (Guthrie & Davis, 2003; Guthrie & Wigfield, 2000). In this context, students tend to view reading as study-related and unpleasurable and not as a recreational activity. As Aschenbeck (1986) notes, “many children leave school without ever suspecting that reading can be a pleasant activity. Our culture has a nervous psyche, an anxiety to get on with things…Schools contribute (to aliteracy) by concentrating on reading for information, not for pleasure or experience” (p. 14). Some assigned reading questions and activities further embarrass and humiliate children and turn them away from reading. Similarly, research has documented a decline in students’ intrinsic motivation due to the competitive and evaluative learning environment in schools, particularly in upper grades (Anderman & Midgley, 1997; Eccles & Midgley, 1989). This environment leads students to spend more time studying than voluntarily reading. For example, Mikulecky (1978) found that early adolescents do little reading other than for assignments. As expected, reading is not always actively encouraged in high schools compared to elementary schools (Wilkinson et al., 2020).

A meta-analysis of early literacy interventions (e.g., providing more diverse reading choices, supporting parent–child literacy interactions, and facilitating children’s self-directed reading and story discussion) found that these improvements in the home environment subsequently resulted in more interest in reading and higher scores on literacy-related skills for children in their early years of school (de Bondt et al., 2020). A similar meta-analysis of research exploring the relationship between print access and learning outcomes found that increased access to print material improves children’s reading outcomes including attitudes toward reading and the amount they read (Lindsay, 2010).

In Lee and Kim’s (2022) study, factors that account for the increases in reading interest include interesting reading materials, improved reading abilities, an autonomous reading environment, school reading programs, and college lectures. By contrast, factors associated with a decline in reading interest include academic burdens and assigned reading, insufficient reading abilities, and insufficient self-regulation abilities regarding digital media use (e.g., smartphones). Other factors such as a lack of time, homework, losing the habit of reading, using a phone or watching television, and difficulty finding a book are also mentioned as barriers to motivating students to read (Wilkinson et al., 2020). In addition, Turner (1992) reported that an inappropriateness or scarcity of materials, lack of reading abilities, past failures in reading, inappropriate instruction, conflicting values on the importance of reading, and a non-reading environment at both home and school contributed to students’ reluctance to read.

Under a narrowly construed understanding of literacy, some research has pointed out that electronic media often dominates young people’s lives (Aschenbeck, 1986). For example, Kim and Heo (2021) revealed that the acquisition of smartphones is one of the main reasons for elementary and middle school students’ aliteracy. It is plausible that smartphone use occupies a majority of teenagers’ leisure time. Therefore, their time for reading is insufficient (Park, 2022). Although digital reading is accepted as a new type of reading that is also important for participating in contemporary life (Leu, 2002), in the educational contexts of Asian countries such as China, digital reading is generally considered *not serious* or just entertainment rather than *real reading* (Li, 2001). Reading is usually bounded by terms such as *serious*, *academic*, and *paper books*.

### 1.3. Chinese Educational Context

In Chinese society, academic success is considered fundamental to securing a respectable social status and satisfactory life (Salili et al., 2001). Chinese parents are generally concerned about their children’s achievements, because obtaining higher grades in school could lead to a better future (Ho, 1986). Given Chinese culture’s emphasis on education as having such a high value, research has found that Chinese students have higher education-related aspirations than Western students (Stevenson, 1992). Owing to the stress of competitive high-stake examinations (e.g., Chinese college entrance examinations), the learning environment in Chinese schools (particularly secondary schools) is highly impersonal, evaluative, and competitive (Lau, 2009). Driven by the competitive examination system, many Chinese language teachers (especially those at the secondary school level) are performance-oriented and rely on drilling and didactic strategies to teach reading (Lau, 2017).

In a typical Chinese language class, teachers assume great responsibility and authority and spend considerable time explaining the content of prescribed texts in textbooks (e.g., background information, vocabulary, and rhetorical use of the texts). Extensive reading, which is important for the growth of reading competence (Anderson, 1996), has largely been neglected, discouraged, and even forbidden because of intense study schedules. Similarly, independent reading has not been emphasized until recently (Yi et al., 2018). The instructional approach that focuses on memorization, drilling, and examination coping strategies seems ill-conceived if the goal of reading instruction is, at least in part, to foster students’ love for reading (Cramer & Castle, 1994). In fact, this instructional approach clashes directly with adolescents’ increasing need for autonomy and self-direction (Eccles & Midgley, 1989). Consequently, Chinese students might find it difficult to develop an intrinsic interest in reading.

### 1.4. The Present Study

Based on the literature discussed above, this study aims to examine how do the reading interest levels of preservice Chinese language teachers change from an early age (e.g., preschool) to college, and what factors have affected their past and current reading interests? Understanding preservice Chinese language teachers’ reading interest profiles and related motivators and barriers are essential for literacy researchers and educators who wish to encourage students to engage in reading and become lifelong readers. Research on this topic is scarce and focuses on other cultural contexts (e.g., South Korea, cf. Lee & Kim, 2022). Therefore, this study seeks to examine the following two questions:

1. What underlying reading interest profiles can be identified among preservice Chinese language teachers?
2. What factors made them begin to like or dislike reading?

## 2. Materials and Methods

### 2.1. Participants

A total of 321 university sophomores (275 women, 85.7%; 46 men, 14.3%; *M*_age_ = 20.28; *SD* = 0.60) at a public university—directed under the management of the Ministry of Education and located in Southeast China—participated in this study. This university is considered a prestigious university as it is one of the 116 universities sponsored by Project 211, which allocates special funds to support the national key universities. Specifically, the participants were drawn from two courses in teacher preparation programs: *Chinese Language Education in Elementary School* (119 students, majoring in *elementary education*) and *Chinese Language Education* (202 students, majoring in *Chinese language and literature education*), both of which are compulsory three-credit core courses. The major of *elementary education* intends to cultivate Chinese language teachers in elementary schools, whereas the major of *Chinese language and literature education* intends to cultivate Chinese language teachers in secondary schools. Informed consent was obtained from all the participants.

### 2.2. Measures

The data used in this study were collected through a reading interest questionnaire (adopted from Lee & Kim, 2022; see Appendix A). The questionnaire contains one close-ended and three open-ended items. The close-ended question asked about participants’ views regarding their reading interests during seven periods of their lives: *preschool*, *1–2 grades*, *3–4 grades*, *5–6 grades*, *middle school*, *high school*, and *college*. Specifically, participants were asked to recall their reading experiences from childhood to the present and mark their reading interest levels in each period using a Likert-style scale with scores ranging from 0 to 10. A higher score indicated a higher level of reading interest. By connecting these points, a reading interest curve was formed. The remaining three open-ended prompts required participants to provide written responses regarding reasons for their particularly high or low reading interest levels and reasons for changes in their reading interest curve. Considering that digital reading such as searching for information on the Internet, text messaging, or watching videos is not considered *real reading* in the Chinese cultural context, in this study, reading was conceptualized as reading paper-based books as well as those read on a computer screen (e.g., static, non-interactive forms such as e-books and PDF files).

### 2.3. Procedure

The survey was administered once in the fall and once in the spring to groups of each major in regular classes. Participants were asked to complete the survey dependently to alleviate concerns that they would alter their responses based on judgment from classmates. Confidentiality was ensured, and students were given full explanations and response examples about the survey questions. One of the researchers moved around the classroom to answer questions as needed. The entire session lasted approximately 25 min.

### 2.4. Analysis

Latent profile analysis. To identify participants’ reading interest profiles, an LPA was conducted using the maximum likelihood (ML) estimator in Mplus 7.4. ML-estimation employs different model parameter estimates using sample data and chooses the estimates associated with the highest likelihood of being gleaned from that sample (Enders, 2005); it is most commonly used to estimate model parameters (Lin et al., 2021). The optimal profile solution should involve models that are consistent with theoretical assumptions, model fit indices, and previous findings (Marsh et al., 2009). To determine the goodness of the model fit, the following indices were used: log likelihood (LL), Akaike information criterion (AIC), Bayesian information criterion (BIC), and sample-size-adjusted BIC (ABIC). The selection of the best-fitting solution was based on higher LL values and lower AIC, BIC, and ABIC values (Nylund-Gibson & Choi, 2018). The Lo-Mendell-Rubin likelihood ratio test (LMRT) (Lo et al., 2001) was employed to evaluate the improvement in the model after adding one latent profile. Significant *p*-values (<0.05) indicated that the model with *k* profiles performed better than that with *k* − 1 profiles (Nylund-Gibson & Choi, 2018). In addition, entropy was used to assess the level of separation between the latent profiles; it falls between 0 and 1, with higher values indicating less classification error (Collins & Lanza, 2010). Concerning the percentage of the sample in each profile of a given model, a model containing profiles with a prevalence below 5% should be rejected (Schiefele & Löweke, 2017).

Qualitative analysis. Qualitative data analysis consisting of coding and categorizing (Miles et al., 2014) was used to analyze written responses to three open-ended questions. First, we transcribed all written responses and became familiar with the data through repeated readings. Descriptive coding was then applied to assign labels to chunks of transcribed data, such as phrases or meaningful statements (e.g., “I was able to recognize most of the words at that time.” [Code: word identification]). The initial codes were then collated into potential higher-order themes (e.g., word identification, comprehension, background knowledge, cognitive development, and empathy [Theme: improved reading ability]). We re-examined the defined themes to ensure comprehensiveness and appropriateness of the coding and categorization of the narratives. Finally, appropriate statements were selected to illustrate the current themes. Statements have been translated from Chinese to English and edited to improve readability, while remaining as close to the original statements as possible. In addition, we counted the number of occurrences of each theme to determine its relative importance. A total of 38.94% of transcripts were randomly selected to be coded by a second trained coder. The average inter-rater reliability was calculated by dividing the number of times the researchers agreed by the total number of units of analysis. The percentage of inter-coder agreement across all files was 95.87%, indicating a high level of inter-rater reliability. In case where the researchers differed in coding, we recoded together until agreement was reached.

## 3. Results and Discussions

### 3.1. Preliminary Analyses

Descriptive statistics and correlations among variables are presented in Table 1. Data distributions are normal. Strong positive correlations exist between reading interests in preschool and elementary schools. Notably, only reading interests in higher grades of elementary school are positively associated with those in secondary schools, and only reading interest in high school is positively associated with that in college.

### 3.2. Reading Interest Profiles of Preservice Chinese Language Teachers

**Figure 1:**
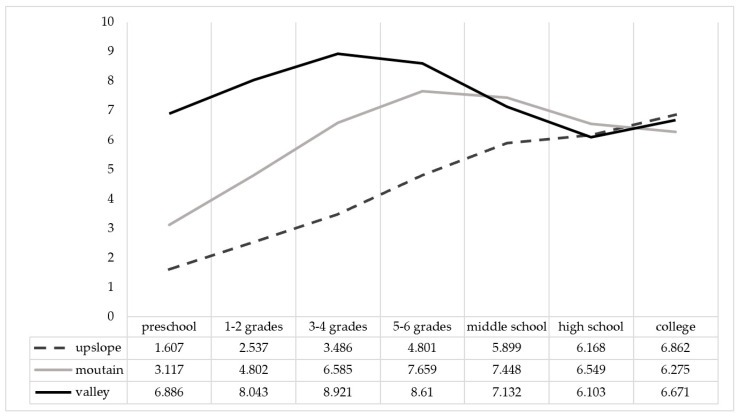
The reading interest profiles among preservice Chinese language teachers.

To identify the best-fitting model, models with one to four profiles were fitted to the available options. Table 2 presents the model fit indices for one to four latent profiles. Specifically, AIC, BIC, and ABIC decreased with each additional profile, suggesting a better fit for more complex solutions. The *p*-value for LMRT revealed that the four-profile model was not significantly better than the three-profile model. Therefore, a three-profile model was selected as the optimal one. Figure 1 shows participants’ reading interest levels during different periods for each profile.

The three profiles contained relatively equal numbers. The *upslope* type (constantly increasing; *n* = 114, 35.5%) showed a substantially low reading interest level at first (*M* = 1.61; the lowest among the three profiles). However, it constantly increased as the students went through higher grades and reached its highest-level during college (*M* = 6.86). The *mountain* type (up-down; *n* = 136, 42.4%) showed a low reading interest level in early childhood (*M* = 3.12). However, it gradually increased (a rising period) and reached the highest level during fifth and sixth grade (*M* = 7.66), after which it began to decline (a declining period). The declining trend in reading interest suggested that this group might be at high risk of becoming non-readers after graduation. Finally, the *valley* type (up-down-up; *n* = 71, 22.1%) showed a high reading interest level at an early age (*M* = 6.89) that continued increasing as students went through the higher grades (a rising period). It reached its peak during third and fourth grade and then started to decline until high school (a declining period). This profile shared many similarities with the *mountain* group, in that both displayed rising and declining periods, and both reached the highest reading interest level in similar time periods. However, compared with the *mountain* type, showing a low starting point and ending with a declining trend, the *valley* type group began with a high reading interest level and again showed an increasing trend after completing high school. These profiles were similar to those reported by Lee and Kim (2022).

All three profiles showed an increasing trend in reading interest from preschool to higher grades in elementary school. This is contrary to previous findings indicating that students’ reading attitudes tend to worsen over time from the lower to upper grades (McKenna et al., 1995). However, the reading interest levels of *valley* and *mountain* profiles started to decline in middle school and reached relatively low levels in high school and college, respectively. This is consistent with Kim’s (2020) finding that high school and ages between 20 and 30 were typical non-reader-formation periods. The *upslope* profile showed an increasing trend from preschool to college despite a substantially low starting point. In addition, only the *mountain* type ended with a declining trend.

### 3.3. Why Did They Begin to Like Reading? The Motivators

**Figure 2:**
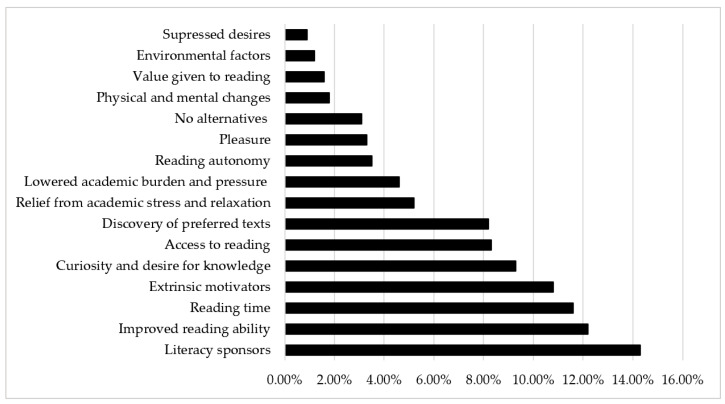
Why they begin to like reading: The motivators.

Figure 2 and Appendix B present the factors affecting the students’ increased reading interests. Many participants mentioned that they began to like reading because of the reading support provided by various *literacy sponsors* (*n* = 177, 14.3%), including *teachers* (*n* = 74, 6.0%), *peers* (*n* = 58, 4.7%), *family members* (*n* = 37, 3.0%), and *school* (*n* = 8, 0.6%). Among these sponsors, teachers (specifically elementary/secondary school teachers and college lecturers) showed the strongest effect on improving students’ reading interests, especially by encouraging reading, providing book recommendations, holding various reading activities, and providing time or opportunities for students to read: “Influenced by my favorite middle school Chinese language teacher, I became interested in reading and writing. Inspired by her, I read *The Romance of the Three Kingdoms* [三国演义], *The Three Heroes and Five Gallants* [三侠五义], and the other required readings for middle school students…Additionally, my high school Chinese language teacher often mentioned his reading experience (e.g., studying ancient Chinese dictionaries overnight, reading extensively from *Records of the Grand Historian* [史记], and reading Russian and Soviet classics). The first million-word-novel I read in college was *And Quiet Flows the Don* [静静的顿河], which was highly recommended by him.” This finding was expected, considering the belief of *respecting teachers and valuing education* [尊师重教], rooted in Confucian culture, as well as the authoritative role teachers play in the Chinese learning context. It also coincides with a previous finding that teachers’ attitude toward reading was relatively transparent to their students (Applegate & Applegate, 2004; Applegate et al., 2014).

However, as students grew older (especially during the higher grades of elementary school and secondary schools), there were more mentions of influences from peers such as direct book recommendations from peers as well as indirect recommendations: “I noticed that my classmates talked about books and authors that I knew very little about. When we talked about that topic, I had nothing to say. I was very curious about the books and authors that my classmates talked about, and I wanted to see what they were like.” Similarly, Nieuwenhuizen (2001) found that some students read so that they would not feel socially excluded from the Harry Potter phenomenon that had enamored their peer group. Especially during secondary school, reading is primarily driven by social motives, such as sharing with peers and gaining their recognition (Merga, 2014; McGeown et al., 2016). After entering college, peers serve more as role models, as one student noted: “People around me enjoyed reading, which continually inspired me.” In addition, literacy sponsorship from family members seemed to be particularly important for cultivating reading interests in the early years of life, as many participants mentioned happy and warm memories of shared reading with their parents at bedtime. Family engagement provides children with reading role models and strengthens the bond between caregivers and students around the activity of reading together. A few participants also mentioned the benefits from reading programs or activities arranged by school, such as reading-related courses and book-sharing activities.

Similarly to previous findings (Lee & Kim, 2022; Turner, 1992; Wilkinson et al., 2020), participants reported that, with *improved reading ability* (*n* = 152, 12.2%), they could read multiple texts, accumulate background knowledge, and successfully read long and difficult books; furthermore, they were able to experience the pleasure and enjoyment from reading. Specifically, participants mentioned *general improvement in reading ability* (*n* = 59, 4.8%) as well as the abilities related to *word identification* (*n* = 45, 3.6%), *comprehension* (*n* = 17, 1.4%), *background knowledge* (*n* = 15, 1.2%), *cognitive development* (*n* = 11, 0.9%), and *empathy* (*n* = 5, 0.4%). As noted by one participant, “With the continuous development of my reading ability, I began to understand and process more complex information. I was no longer satisfied with reading simple storybooks but developed a strong interest in reading materials with depth and breadth.”

Reading ability is an important prerequisite for promoting interest in and motivation to read. Only by understanding the meaning of a book and developing empathy with the characters can one experience the joy of reading, thereby promoting interest in reading other books. This theme was mostly mentioned in three periods: the middle and higher grades in elementary school, high school, and college. In the middle and higher grades in elementary school, participants reported that an increase in literacy, especially the ability to recognize words, enabled them to read more diverse books independently. In high school and college, participants generally mentioned that as their reading ability developed, they could comprehend more complex texts or understand texts more deeply, which they could not understand at a young age: “My interest in reading has increased since high school because, as I grew older, I began to gain new insights into some books that I previously couldn’t really understand.” Similarly, the development of their empathetic abilities enabled them to better understand other people’s emotions and perspectives and thus could better resonate with the reading materials.

*Reading time* (*n* = 144, 11.6%) appeared as another important prerequisite for promoting reading interest: “When I was in elementary school, there were many holidays and I had plenty of time to read.” This category was generally mentioned when explaining the increasing trend of reading interest in elementary school, during which time students had fewer academic burdens and more free time. It was also mostly mentioned during the college period, before which participants had just finished the extremely competitive college entrance examination and could finally feel relaxed. As evidence, participants mentioned a related category of a *lowered academic burdens and pressure* (*n* = 57, 4.6%), which allowed them to spend more time reading: “There was not much academic pressure in elementary school and I had the time and energy to read.”

Consistent with Merga (2017) and Troyer (2017), participants spoke of reasons related to *extrinsic motivators* (*n* = 134, 10.8%), such as *driven by major* (*n* = 61, 4.9%), *academic needs* (*n* = 53, 4.3%), and *self-development* (*n* = 20, 1.6%). The category of *driven by major* was mentioned more by students majoring in *Chinese language and literature education*: “Our professors usually require us to read a lot of books. During the reading process, I discovered my favorite writers like Chuncheng Chen, Zhihan Yang, and Ailing Zhang. I also became interested in their other works and would like to read them.” Participants also mentioned reading for *academic needs*, specifically to enrich one’s knowledge, improve their Chinese language achievement or writing ability, prepare for high school studies, and read classics required by curriculum: “I have read Chinese classics such as *Journey to the West* [西游记] and *Water Margin* [水浒传], which might appear on middle school entrance examinations.” Regarding *self-development*, students noted that they wanted to learn more from books to improve themselves, to enrich their spiritual world, or to seek self-growth. All these statements are related to extrinsic motivators of reading. However, the relationship between intrinsic and extrinsic motivation is complex, and extrinsic motivators have the potential to become internalized (Wilkinson et al., 2020), as cited by participants: “Starting in the upper grades of elementary school, I was forced to read several good books and actually became interested. Then I began to take the initiative in looking for books to read”; “I started to read because the major required extensive reading; however, with professional reading guidance and the recommended reading materials, I gradually discovered the joy of reading.” We could consider using extrinsic motivators as a first step to attracting resistant readers or non-readers to begin reading.

The next motivating factor participants cited frequently was *curiosity and desire for knowledge* (*n* = 116, 9.3%) particularly during the higher grades of elementary school and middle school. Specifically, participants reported that they became interested in reading because of curiosity, the desire to acquire knowledge, to explore the world, or to broaden their horizons. This category mostly appeared in fifth and sixth grades and middle school, in which periods students had developed a certain level of reading ability and were motivated to read to learn: “With an improved literacy level, stronger comprehension ability, and developed self-awareness, I was eager to explore novel worlds and ideas through reading”; “I have a strong desire to acquire knowledge, and books opened up a window for me to understand the outside world.” Just as Doiron (1994) notes: “children are naturally curious, with a great thirst to know about the world around them” (p. 618). Many students read books to learn topics that interested them or to learn about the world (Merga, 2017). Reading allowed them to engage in what Alexander (1997) termed *knowledge-seeking*, an important but neglected area in the study of motivation to read. This code was also characterized as agentic engagement as it “refers to proactive, intentional forms of learning” (Guthrie & Klauda, 2015, p. 42).

Another frequently cited factor is *access to reading* (*n* = 103, 8.3%): “I transferred from a rural elementary school to an urban elementary school; thus, I could access more diverse books.” Specifically, participants mentioned that the book corner in a classroom, school library, city library, or bookstore near their home, as well as reading activities arranged by the school, enabled them to access reading resources, and the exposure to more diverse types of books helped them discover their reading preferences and then improved their interest in reading. This again supported Lindsay’s (2010) finding that increased access to print material improves children’s reading attitudes toward reading. As noted by Bus et al. (2020), access to a diverse array of reading materials not only encourages exploration but also accommodates diverse interests, thus nurturing positive attitudes toward reading. Interestingly, among these statements, *access to reading through digital devices* (e.g., smartphones and computers) seems to be a particularly important way for them to obtain reading resources: “When I got my first smartphone in college, I could easily obtain a massive amount of reading resources such as electronic versions of books from all kinds of apps.” Readers may use online resources to find reading materials that they cannot find in print, for convenience, or simply as an alternative source (Loh & Sun, 2018). Furthermore, this also “reduced the financial burden of buying print books,” as mentioned by a participant. Easily accessible and low-cost Internet access provides students today access to a wide array of literacy materials and activities (Huang et al., 2016). However, accessing books through digital devices needs to be viewed dialectically as it also seems to be a distraction from reading for some participants, which is discussed later.

As another influential factor, the *discovery of preferred texts* (*n* = 102, 8.2%) increased students’ reading interest, particularly during the upper grades of elementary school and middle school: “I discovered my favorite magazine *Yilin Mini Miss* [意林·小小姐] and became fascinated with reading online novels along with detective series like *Tiger Team* [冒险小虎队], *Charlie IX* [查理九世], and *Monster Master* [怪物大师]. Finding books that interested me really opened the door to reading, and it made me eager to explore more.” As students read diverse books, they developed their own reading preferences such as story books, fairy tales, (online) novels, adventure, fantasy, adventure, magazines, and historical books. Considering that situational interest that is limited to a particular book at a specific time might lead to long-term reading motivation (Guthrie et al., 2006), it is important to provide diverse books and help students discover their own reading preferences and dispositions. Related, some participants referred to *pleasure* (*n* = 41, 3.3%) or enjoyment from reading; in those cases, reading was an important form of entertainment: “There were relatively few means of entertainment in high school, and we were only allowed to bring books like novels into school for entertainment. Reading became the main way for us to find pleasure…I read various novels after class, and sometimes I secretly read them during class.” This category mostly appeared in middle and high school periods, during which time students feel enormous academic pressure and tend to consider reading as a way of entertainment. This is a similar finding to the *entertainment and pleasure* code found by Merga (2017). As expected, participants were more likely to obtain pleasure from reading fictional works such as novels.

In a similar context, participants spoke of the *relief from academic stress and relaxation* (*n* = 65, 5.2%), particularly in an extremely competitive and performance-oriented learning context. As expected, this category was mentioned mostly in relation to high school: “There was a lot of academic pressure in high school, and reading helped us relieve stress and relax, and sometimes, it also served as a way to escape learning.” Reading seemed to hold the potential to become a balm or form of escapism, especially when students face challenging moments in school (Wilkinson et al., 2020); for these participants, books provided an occasional “escape from hell” (Nielen, 2016, p. 8). This code is similar to the *relaxation* code found by Merga (2017). Interestingly, “reading was one of the few extracurricular activities that parents approved of, in addition to school coursework”; “During the relatively busy academic years of middle and high school, reading became a form of entertainment that *parents can be rest assured about and teachers agree on*.” With electronic devices being strictly controlled, reading became one of the few ways tacitly approved by parents and teachers for students to relieve academic stress and relax. Similarly to Wilkinson et al.’s (2020) finding, students who read for absorption or escapism were more likely to be drawn to fiction.

Finally, several students referred to the influences of *reading autonomy* (*n* = 43, 3.5%) such as freedom in book selection, few constraints in reading, and no assignments after reading. Furthermore, *no alternatives*, especially no distractions from digital activities (*n* = 39, 3.1%; e.g., “There were no electronic products, and reading books was my only option before bedtime.”), *physical and mental changes*, especially in adolescence (*n* = 22, 1.8%; e.g., “When I was in early adolescence, I started experiencing significant emotional fluctuations. I had strong empathy and could deeply understand characters’ emotions, often placing my own emotions in books.”), *value given to reading* (*n* = 20, 1.6%; e.g., “I became increasingly aware of the importance of reading for personal growth. Books are helpful both for psychological health and for expanding knowledge.”), *environmental factors* (*n* = 15, 1.2%; e.g., “I had transferred to another school, and the reading atmosphere in my new class was good.”), and *suppressed desires* (*n* = 11, 0.9%; e.g., “Reading interest forcibly suppressed during high school sharply increased after entering college.”) were also cited.

### 3.4. Why Did They Begin to Dislike Reading? The Barriers

**Figure 3:**
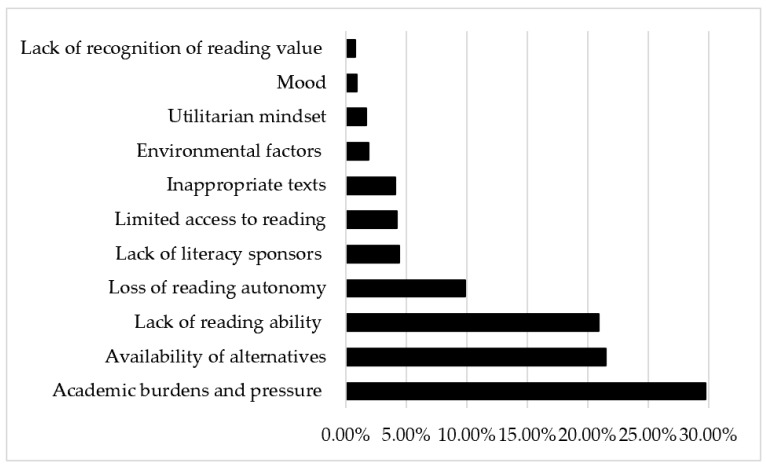
Why they begin to dislike reading: The Barriers.

Figure 3 and Appendix C show the factors affecting students’ decline in reading interest. Several participants reported that their reading interests sharply declined in secondary school, especially in high school, due to heavy *academic burdens and pressure* (*n* = 270, 29.8%). Specifically, they referred to heavy *academic burdens* (*n* = 98, 10.8%; e.g., “The academic burden gradually increased, and brushing through exam questions and engaging in class activities became the focus of my life, leaving no time to read an entire book.”), great *academic pressure* (*n* = 90, 9.9%; e.g., “Under the shadow of the college entrance examination and the pressure of staying in an experimental class, I felt restless and was at a low point every day…I felt depressed, had difficulty concentrating, and became manic whenever I tried to read.”), and then, naturally, *no time to read* (*n* = 51, 5.6%; e.g., “In high school, time was so tight. I could only go home every 14 days, with a lot of homework to complete.”). Even when they had time to read, reading was always performed for *examinations* (*n* = 19, 2.1%; e.g., “In Chinese reading class, I also read magazines helpful for essay writing, accumulate examples and phrases, all for the purpose of exams.”) and for improving *academic achievement* (*n* = 12, 1.3%; e.g., “I focused solely on studying and only wanted to improve my grades. I believed that reading was *ignoring legitimate work* [不务正业] and had little effect on academic performance.”). This theme was mainly mentioned in the *mountain* and *valley* profiles, where students’ reading interests began to decline sharply after entering secondary school.

Owing to the competitive learning environment and heavy academic pressure to enter a good high school and then a prestigious university (Loh & Sun, 2018), Chinese students’ academic burdens and pressure sharply increase during secondary schools, and they must continue engaging in learning activities outside of school. The enjoyable, free, and voluntary reading of children’s literature (e.g., picture books and fairy tales) in elementary school is largely replaced by academic studies (Chong, 2016). This result coincides with previous research demonstrating that a competitive and evaluative learning environment in school, particularly in the upper grades, leads to a decline in students’ intrinsic motivation (Anderman & Midgley, 1997; Eccles & Midgley, 1989). Additionally, reading is not always actively encouraged in high schools compared to elementary schools (Wilkinson et al., 2020). Instead, it is considered to be irrelevant, a waste of time, or a distraction from academic studies: “I had to show my parents that I can maintain a good academic performance even though I spent time reading. I had to make sure that my academic studies were not disturbed by reading.” Similar arguments can also be found in Clark and Rumbold (2006): “reading for pleasure is a ‘cuddly’ activity that some people like to indulge in but that is essentially without further merit” (p. 5). However, this perspective has been challenged by various studies indicating that students who enjoy reading have higher grades in school than those who did not (Mol & Jolles, 2014; Rogiers et al., 2020).

In a typical Chinese secondary school, particularly high school, strict study timetables are imposed, sometimes scheduling every minute for students. The prescriptive study schedule requires students to conduct intensive academic studies, naturally resulting in no time to read. Participants remembered feeling as though their time had been forcibly taken from them. This is consistent with previous findings that high school was a typical non-reader-formation period (Kim, 2020) and that middle adolescence was considered a particularly vulnerable period for reading attitudes and engagement (Clark, 2019). Relatedly, participants mentioned a *utilitarian mindset* (*n* = 15, 1.7%) to indicate that they usually had strong utilitarian reasons and purposefulness regarding reading: “In high school, I had a strong utilitarian mindset related to reading and I mostly read social science books that I didn’t like at all, just for the college entrance examination.”

The next frequently cited factor was *availability of alternatives* (*n* = 195, 21.5%), such as preferring *free play* (e.g., “When I was a child, I preferred playing games with friends rather than reading.”), *watching television* (e.g., “In fifth and sixth grades, I preferred watching television dramas and found it difficult to focus on reading.”), and more commonly, using *digital activities* (e.g., “Just like the famous poem points out, *a riot of blooms begins to dazzle the eye* [乱花渐欲迷人眼]—the online world was too wonderfully distracting.”). These responses indicated that participants chose not to read if other (usually preferable) choices existed, and reading was seen as more effortful than spending time on their phone or watching television (Wilkinson et al., 2020). This finding coincides with studies indicating that students’ interest in reading declines as they become more interested in other activities (e.g., Kirby et al., 2011).

Among these responses, distractions related to digital devices, especially smartphones, were the most commonly mentioned. This mainly accounted for the decline in reading interest from high school to college, as shown in the *mountain* profile. After entering college, almost all the students had their own smartphones, coupled with more free time and a period of psychological relaxation after finishing the college entrance examination. However, owing to their insufficient self-regulation ability, they engaged more in activities such as using social networking services (e.g., WeChat and RedNote), viewing short videos and blogs, and playing online games (see also Lee & Kim, 2022). These activities are generally considered social entertainment activities rather than literacy practices in the Chinese educational context. The rise in technology and its widespread availability seem detrimental to voluntary reading, especially print reading (Aschenbeck, 1986; Kim & Heo, 2021). For many college students, electronic media have largely replaced reading as a leisure activity. Participants also mentioned the side effects of the long-term use of digital devices, especially watching short videos, such as making them increasing impatient (e.g., “I am unable to calm down now. I have been watching too many short videos and am eager to achieve everything in a short time.”) and finding it difficult to concentrate (e.g., “External media such as TikTok and Bilibili have developed rapidly and reached a peak. Short videos have a great impact on reading; for example, they have made it difficult for people to calm down and read.”). This coincides with Hakemulder and Mangen’s (2024) finding that “the more people are exposed to short texts on screen, the less they are inclined to muster the cognitive persistence required for reading a somewhat longer and linear literary text, and to speculate what meaning it might have for them personally” (p. 70).

As Figure 1 shows, all three profiles exhibited the lowest reading interest levels during the first few years of life (e.g., preschool). The main reason cited was the *lack of reading ability* (*n* = 190, 20.9%) of young children. Specifically, the participants not only lacked the ability to *recognize words* (*n* = 79, 8.7%; e.g., “I didn’t know what the book said, I didn’t know the words.”) or *comprehend texts* (*n* = 56, 6.1%; e.g., “Owing to my limited literacy level, it was difficult to understand the content and meaning of books.”), but also lacked *reading awareness* (*n* = 29, 3.2%; e.g., “The concept of reading did not yet exist in my mind at that time.”), *background knowledge* (*n* = 11, 1.2%; e.g., “I had limited knowledge about reading fields and strategies.”), and the ability to *concentrate* (*n* = 15, 1.7%; e.g., “I was a playful preschooler with a relatively short attention span.”). When students do not read well, it is difficult to expect them to experience pleasure and joy from reading and cultivate an interest in reading. Instead, through unsuccessful or unpleasant reading experiences, students might develop a self-defeatist attitude and a negative view of reading (Turner, 1992). Failure in reading makes students feel inferior or incompetent and promoted a negative self-concept (Alexander & Filler, 1976).

Many participants mentioned that as they went through higher grades (e.g., from elementary school to middle and high school), they experienced the *loss of reading autonomy* (*n* = 90, 9.9%). Typically, accountability in reading gradually increased: “Reading was often mandatory and was something that we were forced to do. We were always required to write reading responses, which was time-consuming, labor-intensive, and meaningless.” Furthermore, they experienced the loss of freedom in reading, such as having no voice in deciding what to read and what to do after reading: “My parents and teachers did not support *light reading* or reading *useless books*”; “I underwent significant changes after entering high school, with teachers adopting closed education and basically prohibiting extracurricular reading.” This category is quite similar to Applegate and Applegate’s (2004) finding that students recalled their reading instruction as consisting of “reading dull books” and “doing book reports”. Furthermore, forced academic readings and restricting extracurricular reading not only reduced students’ interest in reading but also distorted their understanding and habits of reading; thus, the negative perception of *reading = learning = boredom* was formed. Participants tended to perceive reading as school- or work-related only, probably because of how it was taught and practiced in the classroom (Turner, 1992). Owing to the extremely competitive learning environment, voluntary reading was almost forbidden in high school; thus, students had to read secretly. As many participants mentioned, they were only permitted to read books in the *to-read list* provided by the school, books matching the curriculum requirements, books considered relevant to academic study (e.g., four Chinese classics, the content of which might appear in reading tests), or books that were considered helpful for improving writing skills and thus would help students achieve higher marks in writing tests. Overall, all reading activities seemed to be examination-oriented, which largely destroyed the students’ inherent interest in reading. A similar situation also happened after they entered college: “After majoring in Chinese language and literature education, my interest in reading has largely decreased. The extracurricular books I used to read became part of the required readings in class. After finishing reading, I mostly focused on completing assignments or writing papers. I no longer just enjoy literature as I did before.”

As barriers to cultivating reading interests in childhood, especially in preschool and early grades in elementary school, some participants cited a *lack of literacy sponsors* (*n* = 40, 4.4%; e.g., “My grandmother raised me until grades 1–2. She had received little education and naturally did not know how to read. My parents, being herdsmen, were away year-round and paid little attention to my studies…The teacher only taught prescribed textbook content and never mentioned we could read books independently.”) and *limited access to reading* (*n* = 38, 4.2%; e.g., “There was no reading corner in the classroom even up to first and second grade, and even if we did have a reading corner later, there were no books available.”). For young children, providing opportunities to access diverse books and sufficient reading support (e.g., parent–child shared reading and book recommendations and discussions) might be important ways to arouse their initial interests in reading.

Participants also noted reasons related to *inappropriate texts* (*n* = 37, 4.1%), such as texts that do not suit their interests (e.g., “There were no books that interested me, and my parents always bought books such as *Tang Poetry* [唐诗], *Stories of Great Minds* [名人故事], and *Grimm’s Fairy Tales* [格林童话].”) as well as texts with high difficulty levels (e.g., “Textbooks and texts recommended by teachers were always classics such as texts written by Xun Lu and Xin Bing. These texts were obscure and really difficult to understand.”). Relevant statements generally appeared during the period from secondary school to college, partly accounting for the decline in reading interests from middle school shown in the *mountain* and *valley* profiles. In a similar context, Mathers and Stern (2012) contend that there is an apparent mismatch between what readers need and the instruction that they receive in the classroom that is said to produce the same result: “Draining the pleasure out of reading” (p. 261). Teachers should be aware of the excellent literature available and use it instead of forcing students to read the classics, which many adolescents are not mature enough to understand (Heins, 1984).

Finally, several participants cited reasons related to *environmental factors* (*n* = 17, 1.9%; e.g., “I entered a high school that was located in an unfamiliar place in Shanghai, and I couldn’t adapt to the fast-paced learning and living environment. I had a lot of study pressure and didn’t want to read.”), *utilitarian mindset* (*n* = 15, 1.7%; e.g., “Under the utilitarian education system, reading was seen as a means to obtain diplomas and achieve career success.”), *mood* (*n* = 8, 0.9%; e.g., “I felt anxious about my life and didn’t feel in the mood to read.”), and *lack of recognition of reading value* (*n* = 7, 0.8%; e.g., “I didn’t understand the importance and significance of reading.”). Although these comments were only made by a few students, it is important to note that they were their spontaneous accounts of why they began to dislike reading.

### 3.5. Profile-Specific Motivators and Barriers

As Figure 1 shows, all three profiles showed increased reading interest levels from preschool to the higher grades of elementary school. Based on participants’ written responses, this increase was mainly influenced by *literacy sponsors* (with the most influential sponsor shifting from parents to teachers and peers as students advanced through higher grades), *improved reading ability*, sufficient *reading time*, *curiosity and desire for knowledge*, *access to reading* materials (primarily print books, e.g., classroom book corners), and the *discovery of preferred texts* (particularly children’s literature such as fairy tales and stories). Regarding the upward trend from middle to high school reflected in the *upslope* profile, key factors included: the *discovery of preferred texts* (especially novels, magazines, historical books, fantasy, and adventure), *extrinsic motivators* (e.g., exam-oriented reading and assigned texts), *physical and mental changes* especially during adolescence, and *no alternatives* (especially no distractions from digital devices). For the post-college increase trend shown in the *upslope* and *valley* profiles, the following factors were cited as main reasons: *access to reading* (especially easy, low-cost Internet access enabling diverse literacy materials and activities), *reading autonomy* (e.g., types of books are no longer limited, as in secondary school, and students have more freedom in choosing what to read), and growing awareness of *reading value*.

Both the *mountain* and *valley* profiles indicated declining reading interest from late elementary to high school. This decrease was attributed to *academic burdens and pressure*, *loss of reading autonomy*, *inappropriate texts*, and *utilitarian mindset*. As adolescents enter secondary school with heavy academic pressure and learning tasks, they began to focus more on improving academic performance, and reading was either considered irrelevant for academic study, or one of the means to improve their academic achievement by reading required materials, resulting in further decrease in reading interests. Regarding the decreasing trend after entering college, as shown by the *mountain* profile, the *availability of alternatives*, especially the unconstrained use of digital devices such as smartphones, was cited as the main type of distraction from reading. This result is consistent with the findings of previous studies showing that college students spend a greater amount of time online today and that digital devices could have a negative impact on the amount of time students devote to conventional reading activities (e.g., Huang et al., 2016).

## 4. Limitations and Educational Implications

### 4.1. Limitations

This study makes an important and unique contribution to the research literature by identifying reading interest profiles and sharing reasons for why preservice Chinese language teachers begin to like or dislike reading in different periods. However, it also had several limitations. First, the sample size was relatively small, and most of the participants were women owing to the characteristics of the education majors. Furthermore, all participants were taking courses that focused on Chinese language education, which might have affected their current interest level in reading. Thus, the generalizability of the findings is limited, and replication studies with larger and more representative samples are strongly recommended.

The second limitation comes from the use of self-reported retrospective data that may cause recall bias. Specifically, the participants were asked to recall and report their reading interest levels during different periods. However, the unreliable nature of memory renders the Likert scores no more than estimates or guesses rather than representations of actual interest levels. More importantly, it might be difficult for participants to recall reading interests at an early age such as in preschool. Instead, their memories from the early years might have been influenced by what their parents had recounted. Thus, longitudinal studies that do not rely on memory as well as questionnaires or interviews targeting parents might be considered supplementary methods for further research.

Third, due to the limited sample size that is hard to split into two random halves, we could not perform a double-split cross-validation procedure to ensure the resulting profile solution was both stable and replicable. In addition, the sample was recruited from only one university, thus there is a risk of a violation of independence. Further research might use a larger and more diverse sample to examine whether similar profile solutions can be identified. In addition, verification mechanisms such as methodological triangulation should be considered. Taken together, the causes of aliteracy are complex, and this study only scratches the surface. In future research, more diverse methods (e.g., case studies and in-depth interviews) are needed to better understand the causes, formation processes, and consequences of aliteracy.

### 4.2. Educational Implications

Despite these limitations, this study has several implications for literacy research and practice. First, although the reading interests of all three profiles gradually increased from early childhood to higher grades in elementary school, there was evidence of decline throughout the three periods of middle school, high school, and, in some cases, college, for different reasons. At the middle school years, all of the profiles began to lose interest in reading (even the *upslope* profile had a lower rate of increase). It appears that this is a critical time for everybody, not just one type of reader or another. In addition, students in the *mountain* profile showed a final declining trend in reading interest, suggesting that they might be at a high risk of becoming non-readers after graduating from college. Based on the motivators and barriers discussed in this study, literacy researchers and educators should consider developing appropriate intervention programs for literacy at different periods.

Second, students may enjoy reading less as they age because of their preoccupation with the increased external academic burden and preparation for impending high-stakes examinations in the Chinese context. Considering that *academic burdens and pressure* are the most influential factors affecting students’ reading interests and that high school is a typical non-reader-formation period, secondary schools should consider how to actively encourage and support adolescents’ reading for pleasure. Initiatives and related instructional practices could be introduced to promote reading for pleasure. For example, several participants who still showed a strong reading interest in highly competitive and stressful learning environments mentioned interesting reading activities held by teachers such as shared reading, poetry reading, book circulation, and the sharing of good books. Chinese teachers should consider employing more motivating instructional practices (e.g., providing choices in texts to be read and connecting students’ interests to reading activities and materials, stimulating peer interactions about texts, and creating a supportive environment) to help students better engage in reading.

Third, our findings suggest that, to better support students’ reading, we must emphasize and listen to their voices to empower them to read. In this study, participants reported that reading fulfilled several needs, such as the need to learn, obtain pleasure, and relax, which are critical to their lives. Therefore, students should have the opportunity to experience the benefits of reading and recognize its power. Guaranteeing regular reading time during the school week, providing books that genuinely resonate with the students or align with their interest and ability level, and most importantly, giving them more autonomy in reading, such as freedom in book selection, including those considered *light reading*, might help achieve this. The last point is particularly important considering that the *loss of reading autonomy* and *inappropriate texts* act as the most influential barriers to students’ reading engagement in addition to *academic burdens and pressure*. In the Chinese educational context, many reading teachers tend to exclusively regard classics as valuable, worth reading, or nutritionally beneficial for student development, without considering text difficulty levels or reader interests. In this context, reading was considered more as a means to improve students’ academic achievement or contribute to their development, thus implying that it is a utilitarian activity, rather than an inherently joyful one.

## Figures and Tables

**Table 1:** Descriptive statistics and correlations among variables.

Reading Interest Level in Different Periods		*M*	*SD*	Skewness	Kurtosis	1	2	3	4	5	6
1	Preschool	3.40	2.52	0.80	−0.15	—					
2	1–2 grades	4.70	2.39	0.32	−0.72	0.82 **	—				
3	3–4 grades	5.99	2.40	−0.06	−0.89	0.64 **	0.80 **	—			
4	5–6 grades	6.84	2.26	−0.49	−0.48	0.40 **	0.54 **	0.76 **	—		
5	Middle school	6.82	2.35	−0.64	−0.19	0.11	0.09	0.27 **	0.42 **	—	
6	High school	6.32	2.48	−0.30	−0.82	−0.07	−0.05	0.01	0.12 *	0.43 **	—
7	college	6.57	2.40	−0.63	−0.18	−0.02	−0.02	−0.09	−0.00	0.01	0.12 *

Note. *M* = Mean, *SD* = Standardized deviation. * *p* < 0.05. ** *p* < 0.01.

**Table 2:** Model fit indices.

Numbers of Profiles	Model Fit Indices							Size of the Smallest Group
	LL	FP	AIC	BIC	ABIC	LMRT (*p*)	Entropy	
1	−5151.154	14	103,30.308	10,383.108	10,338.702	—	—	100.0%
2	−4876.121	22	9796.242	9879.213	9809.433	0.0000	0.860	47.6%
**3**	**−4746.032**	**30**	**9552.065**	**9665.208**	**9570.053**	**0.0010**	**0.867**	**22.1%**
4	−4696.055	38	9468.111	9611.426	9490.896	0.1881	0.864	13.1%

Note. LL, log likelihood; FP, free parameters; AIC, Akaike information criterion; BIC, Bayesian information criterion; ABIC, sample-size adjusted BIC; LMRT, Lo-Mendell-Rubin likelihood ratio test. The bold-faced values represent the model fit indices of the final selected model. LMRT and entropy were not available for the one-profile model.

## Data Availability

The data are not publicly available due to ethical issues.

## Appendix A. Reading Interest Questionnaire

Recall your reading experience from childhood to the present, and draw your reading interest curves in the figure below. *Note: First, identify the period with the highest reading interest and circle its corresponding data dot. Use this point as a benchmark to record the reading interest levels for other periods. Finally, connect all plotted points chronologically to complete the reading interest curve.*									

high low	10								
	9								
	8								
	7								
	6								
	5								
	4								
	3								
	2								
	1								
	0								
		preschool	1–2 grades	3–4 grades	5–6 grades	middle school	high school	college	

Based on the graph, please indicate in which periods did you show the highest and lowest reading interest levels, and what were the possible reasons? In which period did your reading interest level show significant changes, and what factors contribute to this change?									
	*Period with the highest reading interest level and reason:*								

	*Period with the lowest reading interest level and reason:*								

	*Change point in reading interest and reason:*								

## Appendix B. Why Preservice Chinese Language Teachers Begin to Like Reading: The Coding Examples

**Themes**	**Descriptions**	**Examples**
Literacy sponsors (*n* = 177, 14.3%)	Statements indicating literacy support from various sponsors, including *teachers* (*n* = 74, 6.0%), *peers* (*n* = 58, 4.7%), *family members* (*n* = 37, 3.0%), and *school* (*n* = 8, 0.6%).	- The homeroom teacher placed great importance on reading and frequently organized reading activities. She devoted considerable time to students’ reading sessions and established a book corner stocked with various books. - After transferring schools, I met a classmate who had an extensive reading background. She became my role model. - My parents subscribed to numerous journals and magazines for me, and the expanded collection significantly boosted my reading interest. - During this period, the school expanded its library with additional reading materials and literary works, actively encouraging students to explore diverse texts.
Improved reading ability (*n* = 152, 12.2%)	Statements referring to *general improvement in reading ability* (*n* = 59, 4.8%) and those related to *word identification* (*n* = 45, 3.6%), *comprehension* (*n* = 17, 1.4%), *background knowledge* (*n* = 15, 1.2%), *cognitive development* (*n* = 11, 0.9%), and *empathy* (*n* = 5, 0.4%).	- After entering middle school, my cognitive and reading abilities developed considerably, allowing me to comprehend more complex texts. - I acquired more words and reading strategies, which made reading easier and boosted my reading interest. - With improvements in word identification, comprehension skills, and accumulated experiences, my empathy and ability to resonate with texts grew progressively. - My knowledge base expanded, providing a solid foundation for reading. - The transition from elementary school to high school was a small stage of maturity, during which I gained a deeper understanding of certain books, such as classics.
Reading time (*n* = 144, 11.6%)	Sufficient time was guaranteed for reading.	- When I was in elementary school, there were many holidays and I had plenty of time to read - After entering college, I have more free time and can spend it reading books that interest me. - After entering college, academic pressure decreased slightly, so I have more time to explore interests and read books that interest me.
Extrinsic motivators (*n* = 134, 10.8%)	Statements indicating external incentives, including those *driven by major* (*n* = 61, 4.9%), *academic needs* (*n* = 53, 4.3%), and *self-development* (*n* = 20, 1.6%).	- Majoring in Chinese language and literature education has required us to read extensively, whether for coursework or shared-reading activities. This has not only increased my reading volume but also enhanced my interest in reading. - I have read Chinese classics such as *Journey to the West* [西游记] and *Water Margin* [水浒传], which might appear on middle school entrance examinations. - I realize that my literary remains limited and my knowledge needs expansion. I hope to broaden my worldview and enrich my life experience through reading.
Curiosity and desire for knowledge (*n* = 116, 9.3%)	The intrinsic need to learn topics that interested oneself or to learn about the world.	- I was curious about every unknown subject and field. - I have a strong desire to acquire knowledge, and books opened up a window for me to understand the outside world - At this stage (Grades 3–4), I started wanting to explore the world way more. Reading just feel like the best way to do that.
Access to reading (*n* = 103, 8.3%)	Statements referring to *general access to reading* (*n* = 65, 5.2%), *access to more diverse books* (*n* = 18, 1.5%), and *access to reading through digital devices* (*n* = 20, 1.6%)	- There were a lot of cheap books at the convenience store near my school entrance. - After transferring from a rural to an urban elementary school, I suddenly had access to way more diverse books. - When I got my first smartphone in college, I could easily obtain a massive amount of reading resources such as electronic versions of books from all kinds of apps.
Discovery of preferred texts (*n* = 102, 8.2%)	Statements referring to the text types or genres participants preferred, such as story books, fairy tales, (online) novels, adventure, fantasy, magazines, and historical books.	- I discovered my favorite magazine *Yilin Mini Miss* [意林·小小姐] and became fascinated with reading online novels along with detective series like *Tiger Team* [冒险小虎队], *Charlie IX* [查理九世], and *Monster Master* [怪物大师]. Finding books that interested me really opened the door to reading, and it made me eager to explore more. - I devoured children’s classics, animal stories, and fairy tales during that phase. I read extensively and looked forward to seeing a wider world through reading. - During this period, I was exposed to various micro-novels, romance novels, and science fiction. These books strongly appealed to me, who used to be a reluctant reader.
Relief from academic stress and relaxation (*n* = 65, 5.2%)	Reading was regarded as a way to relieve academic stress and achieve relaxation particularly in an extremely competitive and performance-oriented learning context.	- There was a lot of academic pressure in high school, and reading was the main way for me to relieve stress and relax. - Reading was a way to eliminate stress. The busier I was, the more I wanted to read various books. - Academic pressure in high school was extremely high, and reading classical poetry and other similar books could temporarily detach me from reality.
Lowered academic burdens and pressure (*n* = 57, 4.6%)	Statements referring to a lowered academic burdens and pressure that enabled students to have the time and psychological leisure to read.	- There was not much academic pressure in elementary school and I had the time and energy to read - The academic pressure in college was slightly reduced compared to high school. There was more time to explore my interests and hobbies and to read books that interested me. - I was admitted to high school by recommendation, so there was no academic pressure in middle school. I immersed myself in science fiction and romance novels and even wrote a few poems to pass the time.
Pleasure (*n* = 41, 3.3%)	Statements referring to pleasure or enjoyment obtained from reading. Reading became an important form of entertainment especially under great academic pressure.	- There were relatively few means of entertainment in high school, and we were only allowed to bring books like novels into school for entertainment. Reading became the main way for us to find pleasure…I read various novels after class, and sometimes I secretly read them during class. - I once lived in a school dormitory, and during that time reading was my only pleasure. - After obtaining a library card and reading some interesting books, I realized that reading brings genuine happiness.
Reading autonomy (*n* = 43, 3.5%)	More freedom in reading, such as choosing books freely, reading independently, and no assignments after reading.	- Now I have freedom of choice in reading, and I can choose books that I am really interested in. - Although it was stressful and time was tight in high school, I read everything voluntarily, and no one forced me to read. I read many foreign literary classics that I liked. - There was almost no pressure or assignments associated with reading, such as writing a reading response.
No alternatives (*n* = 39, 3.1%)	Statements indicating a lack of other forms of entertainment especially no digital devices available.	- The TV dramas at that time did not suit my taste, so I could only read books to relieve boredom. - There were no electronic products, and reading books was my only option before bedtime. - During high school, there were no electronic devices. In the truly dull study life, extracurricular reading was one of the very few forms of entertainment.
Physical and mental changes (*n* = 22, 1.8%)	Statements referring to physical and mental changes especially entering adolescence, such as emotional fluctuations, mental maturation, and identity seeking.	- When I was in early adolescence, I started experiencing significant emotional fluctuations. I had strong empathy and could deeply understand characters’ emotions, often placing my own emotions in books. - Along with my delicate inner world in middle school, read became my refuge from teenage troubles. - During the emotional and psychological maturation, reading became my tool to seek self-identity, understand others, and explore the world.
Value given to reading (*n* = 20, 1.6%)	Statements indicating the recognition of the value and power of reading.	- I became increasingly aware of the importance of reading for personal growth. Books are helpful both for psychological health and for expanding knowledge. - I read many psychological books such as *The Road Less Travelled* [少有人走的路], which gave me great strength. - I feel very anxious in college, but reading makes me feel happy and calm inside.
Environmental factors (*n* = 15, 1.2%)	Statements referring to external environmental factors, such as changes in learning environment and reading atmosphere.	- I had transferred to another school, and the reading atmosphere in my new class was good. - I was influenced by the academic atmosphere of the department of Chinese language and literature education. - During high school, the school management was relaxed and the reading atmosphere was positive.
Suppressed desires (*n* = 11, 0.9%)	Suppressed desires to read due to a busy academic schedule and high academic pressure were suddenly released.	- Reading interest forcibly suppressed during high school sharply increased after entering college. - After entering middle school, teachers changed from encouraging reading to suppressing it, which was influenced by academic pressure and was thus understandable. However, as the saying goes: “where there is oppression, there is resistance”, the emotions of adolescence that had nowhere to go urgently needed to find an outlet, and reading provided a buffer space.

## Appendix C. Why Preservice Chinese Language Teachers Begin to Dislike Reading: The Coding Examples

**Themes**	**Descriptions**	**Examples**
Academic burdens and pressure (*n* = 270, 29.8%)	Statements that refer to *academic burdens* (*n* = 98, 10.8%), *academic pressure* (*n* = 90, 9.9%), *no time* (*n* = 51, 5.6%), reading for *examinations* (*n* = 19, 2.1%), and reading for *academic achievement* (*n* = 12, 1.3%).	- The academic burden gradually increased, and brushing through exam questions and engaging in class activities became the focus of my life, leaving no time to read an entire book. - Under the shadow of the college entrance examination and the pressure of staying in an experimental class, I felt restless and was at a low point every day…I felt depressed, had difficulty concentrating, and became manic whenever I tried to read. - I had only the college entrance examination on my mind and considered everything else irrelevant and a waste of time. - I focused solely on studying and only wanted to improve my grades. I believed that reading was *ignoring legitimate work* [不务正业] and had little effect on academic performance. - In high school, time was so tight. I could only go home every 14 days and had a massive amount of homework to complete.
Availability of alternatives (*n* = 195, 21.5%)	Availability of other activities for entertainment, including *free play*, *watching TV*, and most importantly, using *digital devices* (*n* = 78, 8.6%).	- When I was a child, I preferred playing games with friends to reading. - In Grades 5–6, I preferred watching television dramas and found it difficult to focus on reading. - Just like the famous poem points out, *a riot of blooms begins to dazzle the eye* [乱花渐欲迷人眼]—the online world was too wonderfully distracting.
Lack of reading ability (*n* = 190, 20.9%)	Statements indicating lack of ability to *recognize words* (*n* = 79, 8.7%), *comprehend texts* (*n* = 56, 6.1%), *concentration* (*n* = 15, 1.7%), lack of *reading awareness* (*n* = 29, 3.2%), and lack of *background knowledge* (*n* = 11, 1.2%).	- I knew too few words to support reading long text and could only read picture books. - The knowledge level during preschool was relatively low, making it difficult to read various types of books. Reading was limited to basic materials such as picture books and stories. - The concept of reading did not yet exist in my mind at that time. - I was a playful preschooler with a relatively short attention span. - I had limited knowledge about reading fields and strategies.
Loss of reading autonomy (*n* = 90, 9.9%)	Statements that refer to loss of freedom in reading, including *required readings* (*n* = 33, 3.6%), *reading accountability* (*n* = 28, 3.1%), *limitations in extracurricular reading materials* (*n* = 16, 1.8%), *teaching approach* (*n* = 7, 0.8%), and *lack of self-selection* (*n* = 6, 0.7%).	- Reading was often mandatory and was something that we were forced to do. We were always required to write reading responses, which was time-consuming, labor-intensive, and meaningless. - My parents and teachers did not support *light reading* or reading *useless books*. - I underwent significant changes after entering high school, with teachers adopting closed education and basically prohibiting extracurricular reading. - The school neither allowed nor recommended reading extracurricular books except for those required in textbooks. - The teacher recommended and introduced essays in class that are “both socialist-minded and professionally proficient”, employing stock phrases like “born under the red flag, growing in the spring winds of reform”, to serve as essay writing templates.
Lack of literacy sponsors (*n* = 40, 4.4%)	Statements that refer to a lack of literacy support from various sponsors, especially parents and teachers.	- My grandmother raised me until grades 1–2. She had received little education and naturally did not know how to read. My parents, being herdsmen, were away year-round and paid little attention to my studies…The teacher only taught prescribed textbook content and never mentioned we could read books independently. - My parents did not focus on cultivating reading habits, so I lacked exposure to diverse reading materials. - There was no systematic arrangement of what books to read, nor did teachers provide guidance on reading.
Limited access to reading (*n* = 38, 4.2%)	Statements that refer to a lack of access to reading resources, such as having no bookstore or library nearby and lacking reading materials at home.	- There was no reading corner in the classroom even up to first and second grade, and even if we did have a reading corner later, there were no books available. - There were too few bookstores around, and buying books was far away and inconvenient. - There were no organized reading activities or extracurricular books in class. Only textbooks were available.
Inappropriate texts (*n* = 37, 4.1%)	Texts that do not match readers’ interests or have an excessively high difficulty level.	- There were no books that interested me, and my parents always bought books such as *Tang Poetry* [唐诗], *Stories of Great Minds* [名人故事], and *Grimm’s Fairy Tales* [格林童话]. - Transferring from a rural kindergarten to an urban elementary school and from being exposed to folk legends and ghost stories to textbooks may be the main reason I lost interest in reading. The picture books and pinyin-annotated Chinese books in elementary school were far inferior to stories like *Strange Stories from a Chinese Studio* [聊斋志异] and *The Romance of the Three Kingdoms* [三国演义] that my grandfather told me. - Textbooks and most teacher recommendations are classics, which are obscure and difficult to understand.
Environmental factors (*n* = 17, 1.9%)	Statements that refer to external environmental factors, such as changes in learning environment and reading atmosphere.	- I entered a high school located in an unfamiliar place in Shanghai, and I couldn’t adapt to the fast-paced learning and living environment. I had a lot of study pressure and didn’t want to read. - I was busy adapting to new environments and making new friends, thus temporarily putting reading on the back burner. - My high school boarding life restricted my reading activities.
Utilitarian mindset (*n* = 15, 1.7%)	Reading is regarded as a means of achieving academic success or professional advancement.	- Under the utilitarian education system, reading was seen as a means to obtain diplomas and achieve career success. - In high school, I had a strong utilitarian mindset in reading. I mostly read social science books that I didn’t like, just for the college entrance examination. - The reading assigned by the school was to read current events for writing, which was too utilitarian.
Mood (*n* = 8, 0.9%)	Statements indicating the lack of mental conditions to read.	- I felt anxious and was unable to get down to reading. - I felt anxious about my life and didn’t feel in the mood to read. - Under the shadow of the college entrance examination and the pressure of staying in an experimental class, I felt restless and at a low point every day. I didn’t have the mood to read.
Lack of recognition of reading value (*n* = 7, 0.8%)	Statements indicating a lack of awareness or recognition of the value and power of reading.	- I didn’t understand the importance and significance of reading. - I did not seriously think about reading at that time, and reading was not considered necessary.
